# Supplementary material for: Genomic Space of MGMT in Human Glioma Revisited: Novel Motifs, Regulatory RNAs, NRF1, 2, and CTCF Involvement in Gene Expression
Source: Int J Mol Sci. 2021 Mar 2;22(5):2492. doi: 10.3390/ijms22052492 (PMC7958331; doi:10.3390/ijms22052492)
Supplement: Supplementary file 1 [file ijms-22-02492-s001.pdf]

Supplementary Information for the Article "Genomic space of MGMT in Human Glioma Revisited: Novel Motifs, Regulatory RNAs, NRF1, 2 and CTCF Involvement in Gene Expression"

Supplementary Tables and Figures are listed in the order of their appearance in the article.

Table S1. Sequences and locations of the five exons of human *MGMT* NM_002412 mRNA (1372 bps). Bold letters show the coding sequence (CDS) and translated regions of the exons reported by the GenBank-Nucleotide. The italic bold letters indicate the translated CDS reported in Uniport and Enzyme Portal. Map positions are shown on the forward strand of chromosome 10/hg38.

MGMT mRNA	Sequences of MGMT exons	Span	Map location	Notes
**Exon 1**	**AACGCTTTGCGTCCCGACGCCCGCAGGTC<br>CTCGCGGTGCGCACCGTTTGCGACTTG**	**1-56<br>(56 bp)**	**129467241-129467296**	**Not translated**
**Exon 2<br>Region A**	**GTACTTGGAAAA**	**57-68<br>(12 bp)**	**129536241-129536252**	**Not translated**
**Exon 2**	***ATGGACAAGGATTGTGAAATGAAACGCA<br>CCACACTGGACAGCCCTTTGGGGAAGCTG<br>GAGCTGTCTGGTTGTGAGCAGGGTCTGCA<br>CGAAATAAAGCTCCTGGGCAAGGGGACG<br>TCTGCAGCTGA***	**69-193 (126 bp)**	**129536253-129536377**	**Translated**
**Exon 3**	***TGCCGTGGAGGTCCCAGCCCCCGCTGCGG<br>TTCTCGGAGGTCCGGAGCCCCTGATGCAG<br>TGCACAGCCTGGCTGAATGCCTATTTCCAC<br>CAGCCCGAGGCTATCGAAGAGTTCCCCGT<br>GCCGGCTCTTCACCATCCCGTTTTCCAGCA<br>AG***	**194-342<br>(149 bp)**	**129707895-129708043**	**Translated**
**Exon 4**	***AGTCGTTCACCAGACAGGTGTTATGGAAG<br>CTGCTGAAGGTTGTGAAATTCGGAGAAGT<br>GATTTCTTACCAGCAATTAGCAGCCCTGGC<br>AGGCAACCCCAAAGCCGCGCGAGCAGTG<br>GGAGGAGCAATGAGAGGCAATCCT***	**343-482<br>(140 bp)**	**129759202-129759341**	**Translated**
**Exon 5<br>Region A**	***GTCCCCATCCTCATCCCGTGCCACAGAGTG<br>GTCTGCAGCAGCGGAGCCGTGGGCAACTA<br>CTCCGGAGGACTGGCCGTGAAGGAATGG<br>CTTCTGGCCCATGAAGGCCACCGGTTGGG<br>GAAGCCAGGCTTGGGAGGGAGCTCAGGT<br>CTGGCAGGGGCCTGGCTCAAGGGAGCGG<br>GAGCTACCTCGGGCTCCCCGCCTGCTGGCC<br>GAAACTGA***	**483-692<br>(210 bp)**	**129766788-129766997**	**Translated**
**Exon 5<br>Region B**	**GTATGTGCAGTAGGATGGATG…ATTTGA<br>TTAAAAGTTTGTGTTTAAAGA**	**693-4678<br>(3986 bp)**	**129766998-129770983**	**Not translated**

```
GGATCCTGCT CCCTCTGAAG GCTCCAGGGA AGAGTGTCCT CTGCTCCCTC  50
CGAAGGCTCC AGGGAAGGGT CTGTCCTCTT AGGCTTCTGG TGGCTTGCAG  100
GTGCAGCCCT CCAATCCTCC TCCCCAAGCG GCCTTCTGCC TATAAGGACA  150
CGAGTCATAC TGGATGAGGG GCCCACTAAT TGATGGCTTC TGTAAAGTCC  200
CCATCTCCAA ATAAGGTCAC ATTGTGAGGT ACTGGGAGTT AGGACTCCAA  250
CATAGCTTCT CTGGTGGACA CAATTCAACT CCTAATAACG TCCACACAAC  300
CCCAAGCAGG GCCTGGCACC CTGTGTGCTC TCTGGAGAGC GGCTGAGTCA  350
GGCTCTGGCA GTGTCTAGGC CATCGGTGAC TGCAGCCCCT GGACGGCATC  400
GCCCACCACA GGCCCTGGAG GCTGCCCCCA CGGCCCCCTG ACAGGGTCTC  450
TGCTGGTCTG GGGGTCCCTG ACTAGGGGAG CGGCACCAGG AGGGGAGAGA  500
CTCGCGCTCC GGGCTCAGCG TAGCCGCCCC GAGCAGGACC GGGATTCTCA  550
CTAAGCGGGC GCCGTCCTAC GACCCCCGCG CGCTTTCAGG ACCACTCGGG  600
CACGTGGCAG GTCGCTTGCA CGCCCGCGGA CTATCCCTGT GACAGGAAAA  650
GGTACGGGCC ATTTGGCAAA CTAAGGCACA GAGCCTCAGG CGGAAGCTGG  700
GAAGGCGCCG CCCGGCTTGT ACCGGCCGAA GGGCCATCCG GGTCAGGCGC  750
ACAGGGCAGC GGCGCTGCCG GAGGACCAGG GCCGGCGTGC CGGCGTCCAG  800
CGAGGATGCG CAGACTGCCT CAGGCCCGGC GCCGCCGCAC AGGGCATGCG  850
CCGACCCGGT CGGGCGGGAA CACCCCGCCC CTCCCGGGCT CCGCCCCAGC  900
TCCGCCCCCG CGCGCCCCGG CCCCGCCCCC GCGCGCTCTC TTGCTTTTCT  950
CAGGTCCTCG GCTCCGCCCC GCTCTAGACC CCGCCCCACG CCGCCATCCC  1000
CGTGCCCCTC GGCCCCGCCC CCGCGCCCCG GATATGCTGG GACAGCCCGC  1050
GCCCCTAGAA CGCTTTGCGT CCCGACGCCC GCAGGTCCTC GCGGTGCGCA  1100
CCGTTTGCGA CTTGGTGAGT GTCTGGGTCG CCTCGCTCCC GGAAGAGTGC  1150
GGAGCTCTCC CTCGGGACGG TGGCAGCCTC GAGTGGTCCT GCAGGCGCCC  1200
TCACTTCGCC GTCGGGTGTG GGGCCGCCCT GACCCCCACC CATCCCGGGC  1250
GAGCTCCAGG TGCGCCCCAA GTGCCTCCCA GGTGTTGCCC AGCCTTTCCC  1300
CGGGCCTGGG GTTCCTGGAC TAGGCTGCGC TGCAGTGACT GTGGACTGGC  1350
GTGTGGCGGG GGTCGTGGCA GCCCCTGCCT TACCTCTAGG TGCCAGCCCC  1400
AGGCCCGGGC CCCGGGTTCT TCCTACCCTT CCATGCTGCC AGCTTTCCCT  1450
CCGCCAGCTG CTCCAGGAAG CTTCCAGAAG CCCCTGCGCG GGCCTTGGCT  1500
TGCAGCAACC CTTTAGCATA CTTAGGCAGA GTCCCATATT TCCTTCCTGC  1550
TGGAGGCCAA GTTCTAGGGG CCTTCTGGTT ACTATGGCTG GTGTTTGTGT  1600
ACATCATACC CTAACTGTAT TCATCAACAC TTAGAGTAAG CAAGGCTCGC  1650
TGGAGAGCCA CACACACTGG GCACCGTAAT GTCGGTTATA ACACCGCAGA  1700
GGAGTTCTGA ACTATGTATT TCGCACTCCT GGGTTCATCA TCTCCTGAAA  1750
TCTCAGGGTG GTGTTTGCTC TCAGTTGCTT CAGCTGAGTA GCTGGCTTTC  1800
TGTCCTGGAA AGCAGACTTT GTACATGTGT GTGCAACCTA TGCCTGCTGA  1850
GATCATCATC AGACAGGGAA GCGGCTTGGT CCAGAGAGCT GTTCTCAGTA  1900
GAATGTTAAG CACAGAGAGC TGAGAATTAG ACTGGTTATT TACATAGACA  1950
TCCAAATAGA AACCTATAGA GTATCTGTTA AGTCAGGCTC TCCCGTCATC  2000
TCCCCCATCC CTGGGCAGG                                    2019
```

Figure S1. Revised *MGMT* promoter of 2019 bp as reported in this study. The X61657.1 minimal promoter sequence described by Harris et. al., 1991 [23] is shown in red. The promoter was further extended by 862 bp to include five overlapping alternative promoters published in different databases. Untranslated MGMT exon 1 is underlined.

EPD: https://epd.epfl.ch/cgi-bin/get_doc?db=hgEpdNew&format=genome&entry=MGMT_1

Ensembl 85: Jul 2016:
http://jul2016.archive.ensembl.org/Homo_sapiens/Gene/Regulation?db=core;g=ENSG00000170430;r=10:129467184-129768007

Ensembl 100: Apr 2020:
http://useast.ensembl.org/Homo_sapiens/Gene/Regulation?db=core;g=ENSG00000170430;r=10:129467190-129770983

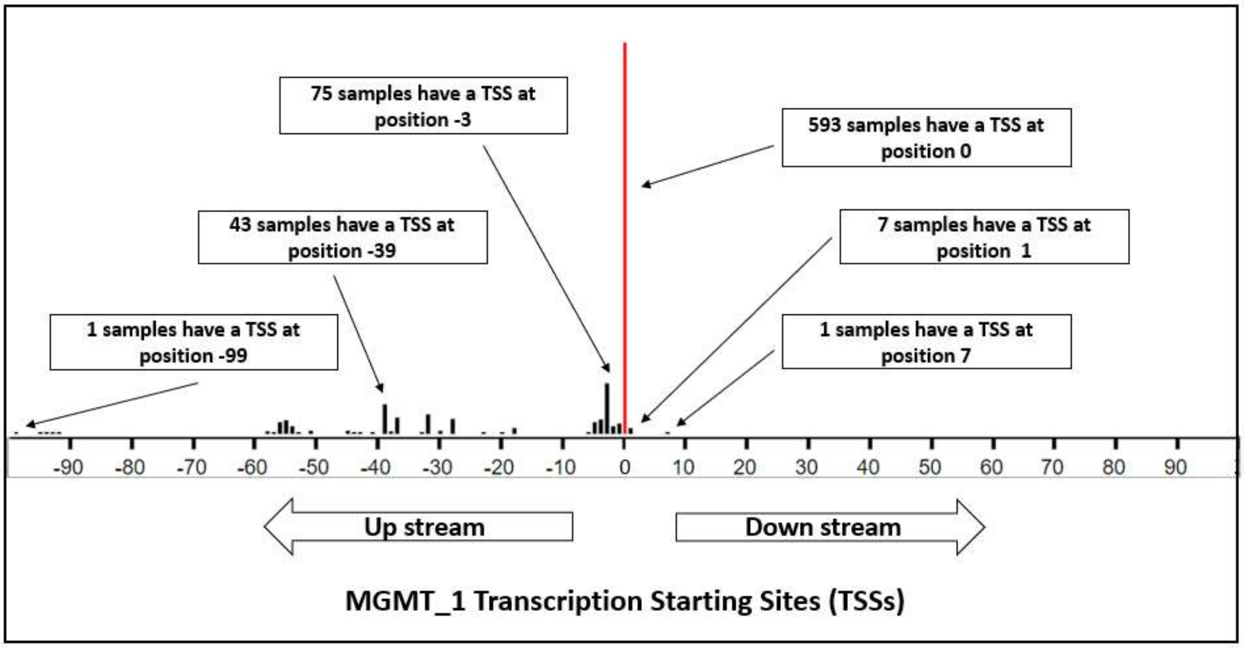

Figure S2. Transcription starting sites in *MGMT*_1 promoter as reported in EPD promoter database. MGMT_1 is expressed in 930 cells and tissues samples with an average expression of 125.099 tags per 10M. 593 samples have a TSS at position 0. Two TSSs were identified downstream: 7 samples had a TSS at position 1, and 1 sample had a TSS at position 7. Thirty-two TSSs identified upstream, e.g., 5 samples have a TSS at position -3, 21 samples have a TSS at position -28, and 43 samples had a TSS at position -39.

(Ref.EPD: https://epd.epfl.ch/cgi/bin/get_doc?db=hgEpdNew&format=genome&entry=MGMT_1).

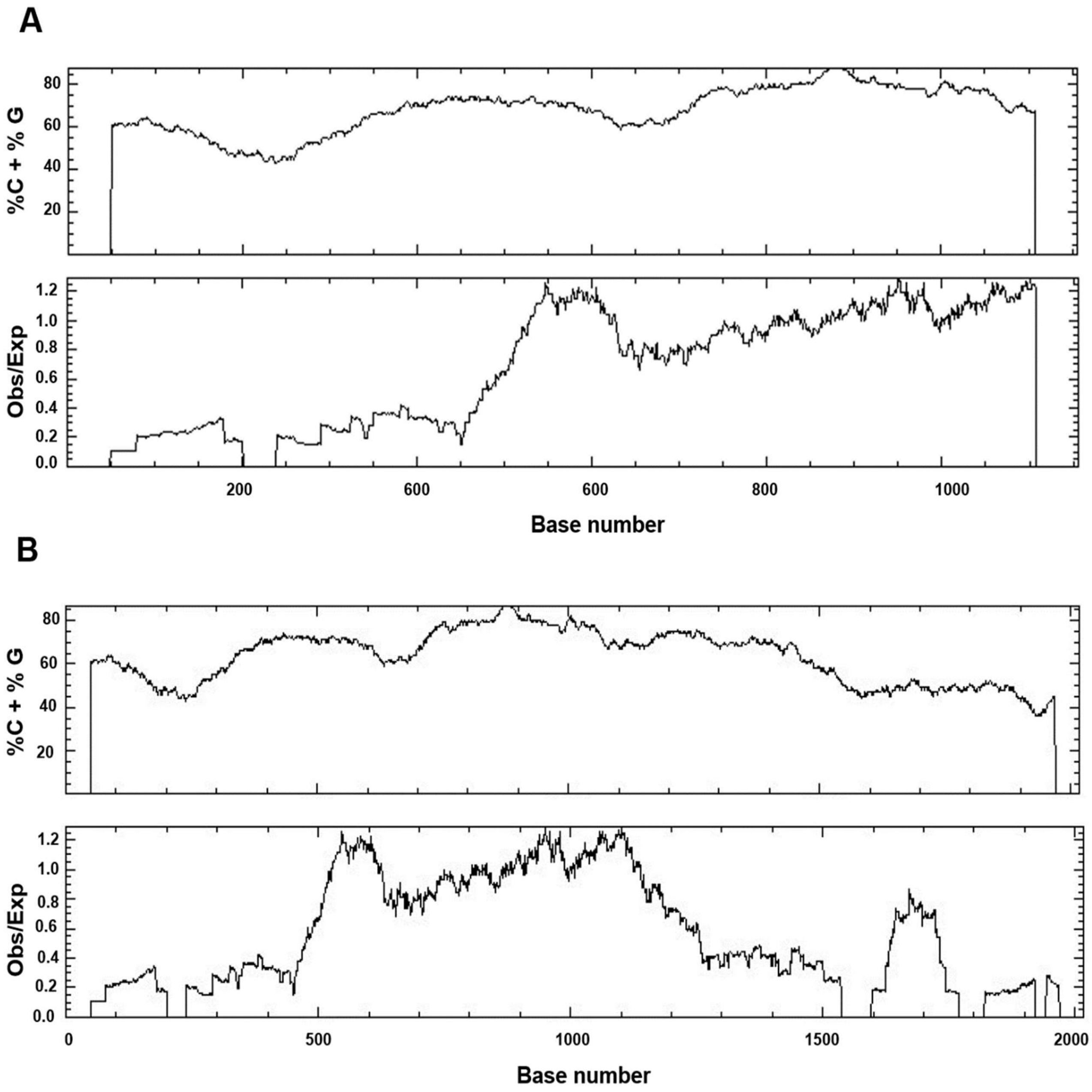

Figure S3. The CGI plots of *MGMT* promoter. A - The original X61657.1 sequence reported in the GenBank-nucleotide is composed of 1157 bps and hosts CGI of 621 bps (481-1101). The MGMT-P1 revised version of 2019 bps promoter hosts CGI of 776 bps (476-1251). CGIs parameters: Observed/Expected ratio > 0.60, Percent C + Percent G > 50.00, Length > 200 bps.

Table S2. Genomic context of siRNA and shRNA used in this study that target the *CTCF* transcript variant 1 sequence NM_006565.3. The map location of the target sequences mapped at the CTCF transcript (NM_006565), and CTCF genomic space (Gene ID: 10664), chr16: 67562407-67639185. E3, E5, E7, E8 and E12 indicate the exons of *CTCF* transcript.

Type	Code	length	Target sequence	Location in the *CTCF* transcript	Location in the CTCF genomic space at chr16, + strand
siRNA	SR307273A	25	GCAGTGTACAGATGGTGATG ATGGA	611-635 (E3)	67610999-67611023
	SR307273B	25	GCATTTGAACCTTGTATAATTA ACT	3416-3440 (E12)	67638660-67638684
	SR307273C	25	GCTGTACAGCTAATAAATCAT AACG	3896-3920 (E12)	67639140-67639164
shRNA	HSH000809-1	19	GTGACTGTACCTGTTGCTA	808-826 (E3)	67611196-67611214
	HSH000809-2	19	ATCGTCGTTACAAACACAC	1463-1481 (E5)	67616811-67616829
	HSH000809-3	19	ATGTGGCCAAATTTCACTG	1742-1760 (E7)	67621532-67621550
	HSH000809-4	19	GACCAGTGTGATTACGCTT	1936-1954 (E8)	67626689-67626707
